# Supplementary material for: Pyruvate Dehydrogenase Kinase 1 inhibition mediated oxidative phosphorylation enhancement in cartilage promotes osteoarthritis progression
Source: BMC Musculoskelet Disord. 2023 Jul 20;24:597. doi: 10.1186/s12891-023-06585-6 (PMC10357736; doi:10.1186/s12891-023-06585-6)
Supplement: Supplementary file 1 — Additional file 1. [file 12891_2023_6585_MOESM1_ESM.zip › MF.Gsea.1653627316242/GOMF_NUCLEAR_RECEPTOR_BINDING.html]

Details for gene set GOMF\_NUCLEAR\_RECEPTOR\_BINDING[GSEA]

|  || Dataset | 55457\_collapsed\_to\_symbols.cls.cls #OA\_versus\_normal.cls.cls #OA\_versus\_normal\_repos |
| Phenotype | cls.cls#OA\_versus\_normal\_repos |
| Upregulated in class | normal |
| GeneSet | GOMF\_NUCLEAR\_RECEPTOR\_BINDING |
| Enrichment Score (ES) | -0.31858137 |
| Normalized Enrichment Score (NES) | -1.4470112 |
| Nominal p-value | 0.0060698027 |
| FDR q-value | 0.23165907 |
| FWER p-Value | 1.0 |
Table: GSEA Results Summary

  

Fig 1: Enrichment plot: GOMF\_NUCLEAR\_RECEPTOR\_BINDING      
 Profile of the Running ES Score & Positions of GeneSet Members on the Rank Ordered List

  

| SYMBOL | TITLE | RANK IN GENE LIST | RANK METRIC SCORE | RUNNING ES | CORE ENRICHMENT || 1 | TRIM68 | tripartite motif containing 68 [Source:HGNC Symbol;Acc:HGNC:21161] | 34 | 1.023 | 0.0205 | No |
| 2 | RNF14 | ring finger protein 14 [Source:HGNC Symbol;Acc:HGNC:10058] | 90 | 0.926 | 0.0371 | No |
| 3 | YWHAH | tyrosine 3-monooxygenase/tryptophan 5-monooxygenase activation protein eta [Source:HGNC Symbol;Acc:HGNC:12853] | 118 | 0.889 | 0.0551 | No |
| 4 | SLC30A9 | solute carrier family 30 member 9 [Source:HGNC Symbol;Acc:HGNC:1329] | 390 | 0.710 | 0.0499 | No |
| 5 | TRIP6 | thyroid hormone receptor interactor 6 [Source:HGNC Symbol;Acc:HGNC:12311] | 524 | 0.650 | 0.0542 | No |
| 6 | NR0B2 | nuclear receptor subfamily 0 group B member 2 [Source:HGNC Symbol;Acc:HGNC:7961] | 573 | 0.632 | 0.0647 | No |
| 7 | ESR1 | estrogen receptor 1 [Source:HGNC Symbol;Acc:HGNC:3467] | 984 | 0.522 | 0.0444 | No |
| 8 | PRMT2 | protein arginine methyltransferase 2 [Source:HGNC Symbol;Acc:HGNC:5186] | 1171 | 0.482 | 0.0408 | No |
| 9 | TADA3 | transcriptional adaptor 3 [Source:HGNC Symbol;Acc:HGNC:19422] | 1295 | 0.459 | 0.0415 | No |
| 10 | GTF2B | general transcription factor IIB [Source:HGNC Symbol;Acc:HGNC:4648] | 1315 | 0.455 | 0.0503 | No |
| 11 | WBP2 | WW domain binding protein 2 [Source:HGNC Symbol;Acc:HGNC:12738] | 1344 | 0.451 | 0.0583 | No |
| 12 | BUD31 | BUD31 homolog [Source:HGNC Symbol;Acc:HGNC:29629] | 1380 | 0.446 | 0.0657 | No |
| 13 | PARK7 | Parkinsonism associated deglycase [Source:HGNC Symbol;Acc:HGNC:16369] | 1406 | 0.442 | 0.0737 | No |
| 14 | BCAS3 | BCAS3 microtubule associated cell migration factor [Source:HGNC Symbol;Acc:HGNC:14347] | 1471 | 0.433 | 0.0785 | No |
| 15 | PCNA | proliferating cell nuclear antigen [Source:HGNC Symbol;Acc:HGNC:8729] | 1557 | 0.419 | 0.0813 | No |
| 16 | ZNHIT3 | zinc finger HIT-type containing 3 [Source:HGNC Symbol;Acc:HGNC:12309] | 1648 | 0.407 | 0.0834 | No |
| 17 | C1D | C1D nuclear receptor corepressor [Source:HGNC Symbol;Acc:HGNC:29911] | 1848 | 0.379 | 0.0764 | No |
| 18 | ISL1 | ISL LIM homeobox 1 [Source:HGNC Symbol;Acc:HGNC:6132] | 2373 | 0.318 | 0.0426 | No |
| 19 | PKN1 | protein kinase N1 [Source:HGNC Symbol;Acc:HGNC:9405] | 2540 | 0.298 | 0.0363 | No |
| 20 | NCOR1 | nuclear receptor corepressor 1 [Source:HGNC Symbol;Acc:HGNC:7672] | 2549 | 0.297 | 0.0424 | No |
| 21 | MED12 | mediator complex subunit 12 [Source:HGNC Symbol;Acc:HGNC:11957] | 2588 | 0.292 | 0.0461 | No |
| 22 | PADI2 | peptidyl arginine deiminase 2 [Source:HGNC Symbol;Acc:HGNC:18341] | 2810 | 0.269 | 0.0348 | No |
| 23 | TAF7 | TATA-box binding protein associated factor 7 [Source:HGNC Symbol;Acc:HGNC:11541] | 3035 | 0.246 | 0.0229 | No |
| 24 | GAS2L1 | growth arrest specific 2 like 1 [Source:HGNC Symbol;Acc:HGNC:16955] | 3136 | 0.237 | 0.0204 | No |
| 25 | PRAME | PRAME nuclear receptor transcriptional regulator [Source:HGNC Symbol;Acc:HGNC:9336] | 3235 | 0.227 | 0.0178 | No |
| 26 | MED24 | mediator complex subunit 24 [Source:HGNC Symbol;Acc:HGNC:22963] | 3246 | 0.226 | 0.0222 | No |
| 27 | LRIF1 | ligand dependent nuclear receptor interacting factor 1 [Source:HGNC Symbol;Acc:HGNC:30299] | 3284 | 0.222 | 0.0243 | No |
| 28 | PARP1 | poly(ADP-ribose) polymerase 1 [Source:HGNC Symbol;Acc:HGNC:270] | 3410 | 0.210 | 0.0193 | No |
| 29 | VDR | vitamin D receptor [Source:HGNC Symbol;Acc:HGNC:12679] | 3571 | 0.197 | 0.0112 | No |
| 30 | TGFB1I1 | transforming growth factor beta 1 induced transcript 1 [Source:HGNC Symbol;Acc:HGNC:11767] | 3628 | 0.191 | 0.0111 | No |
| 31 | HMGN3 | high mobility group nucleosomal binding domain 3 [Source:HGNC Symbol;Acc:HGNC:12312] | 3818 | 0.174 | 0.0002 | No |
| 32 | CNOT1 | CCR4-NOT transcription complex subunit 1 [Source:HGNC Symbol;Acc:HGNC:7877] | 3842 | 0.172 | 0.0023 | No |
| 33 | NR1I2 | nuclear receptor subfamily 1 group I member 2 [Source:HGNC Symbol;Acc:HGNC:7968] | 3956 | 0.160 | -0.0029 | No |
| 34 | SMAD3 | SMAD family member 3 [Source:HGNC Symbol;Acc:HGNC:6769] | 4061 | 0.150 | -0.0077 | No |
| 35 | TRIP4 | thyroid hormone receptor interactor 4 [Source:HGNC Symbol;Acc:HGNC:12310] | 4105 | 0.146 | -0.0077 | No |
| 36 | WIPI1 | "WD repeat domain, phosphoinositide interacting 1 [Source:HGNC Symbol;Acc:HGNC:25471]" | 4188 | 0.139 | -0.0110 | No |
| 37 | SMARCE1 | "SWI/SNF related, matrix associated, actin dependent regulator of chromatin, subfamily e, member 1 [Source:HGNC Symbol;Acc:HGNC:11109]" | 4255 | 0.132 | -0.0132 | No |
| 38 | NCOA2 | nuclear receptor coactivator 2 [Source:HGNC Symbol;Acc:HGNC:7669] | 4284 | 0.129 | -0.0125 | No |
| 39 | PROX1 | prospero homeobox 1 [Source:HGNC Symbol;Acc:HGNC:9459] | 4299 | 0.128 | -0.0106 | No |
| 40 | TCF21 | transcription factor 21 [Source:HGNC Symbol;Acc:HGNC:11632] | 4321 | 0.127 | -0.0094 | No |
| 41 | PHB2 | prohibitin 2 [Source:HGNC Symbol;Acc:HGNC:30306] | 4354 | 0.124 | -0.0091 | No |
| 42 | STAT1 | signal transducer and activator of transcription 1 [Source:HGNC Symbol;Acc:HGNC:11362] | 4495 | 0.113 | -0.0175 | No |
| 43 | PRPF6 | pre-mRNA processing factor 6 [Source:HGNC Symbol;Acc:HGNC:15860] | 4567 | 0.108 | -0.0207 | No |
| 44 | FLT3 | fms related receptor tyrosine kinase 3 [Source:HGNC Symbol;Acc:HGNC:3765] | 4657 | 0.099 | -0.0254 | No |
| 45 | OASL | 2'-5'-oligoadenylate synthetase like [Source:HGNC Symbol;Acc:HGNC:8090] | 4812 | 0.086 | -0.0355 | No |
| 46 | PPID | peptidylprolyl isomerase D [Source:HGNC Symbol;Acc:HGNC:9257] | 5029 | 0.071 | -0.0508 | No |
| 47 | TCF7L2 | transcription factor 7 like 2 [Source:HGNC Symbol;Acc:HGNC:11641] | 5034 | 0.070 | -0.0495 | No |
| 48 | SMARCD3 | "SWI/SNF related, matrix associated, actin dependent regulator of chromatin, subfamily d, member 3 [Source:HGNC Symbol;Acc:HGNC:11108]" | 5036 | 0.070 | -0.0480 | No |
| 49 | CTBP2 | C-terminal binding protein 2 [Source:HGNC Symbol;Acc:HGNC:2495] | 5066 | 0.067 | -0.0488 | No |
| 50 | NSD1 | nuclear receptor binding SET domain protein 1 [Source:HGNC Symbol;Acc:HGNC:14234] | 5178 | 0.058 | -0.0562 | No |
| 51 | MED17 | mediator complex subunit 17 [Source:HGNC Symbol;Acc:HGNC:2375] | 5257 | 0.052 | -0.0611 | No |
| 52 | NR1H2 | nuclear receptor subfamily 1 group H member 2 [Source:HGNC Symbol;Acc:HGNC:7965] | 5413 | 0.039 | -0.0724 | No |
| 53 | SNW1 | SNW domain containing 1 [Source:HGNC Symbol;Acc:HGNC:16696] | 5472 | 0.035 | -0.0761 | No |
| 54 | STAT5B | signal transducer and activator of transcription 5B [Source:HGNC Symbol;Acc:HGNC:11367] | 5554 | 0.028 | -0.0818 | No |
| 55 | ARID1A | AT-rich interaction domain 1A [Source:HGNC Symbol;Acc:HGNC:11110] | 5735 | 0.013 | -0.0956 | No |
| 56 | MED14 | mediator complex subunit 14 [Source:HGNC Symbol;Acc:HGNC:2370] | 5867 | 0.004 | -0.1058 | No |
| 57 | PPARG | peroxisome proliferator activated receptor gamma [Source:HGNC Symbol;Acc:HGNC:9236] | 6006 | -0.005 | -0.1165 | No |
| 58 | FOXL2 | forkhead box L2 [Source:HGNC Symbol;Acc:HGNC:1092] | 6068 | -0.009 | -0.1210 | No |
| 59 | PPARGC1B | PPARG coactivator 1 beta [Source:HGNC Symbol;Acc:HGNC:30022] | 6184 | -0.019 | -0.1296 | No |
| 60 | MED16 | mediator complex subunit 16 [Source:HGNC Symbol;Acc:HGNC:17556] | 6240 | -0.023 | -0.1334 | No |
| 61 | PRKCB | protein kinase C beta [Source:HGNC Symbol;Acc:HGNC:9395] | 6453 | -0.039 | -0.1491 | No |
| 62 | NCOR2 | nuclear receptor corepressor 2 [Source:HGNC Symbol;Acc:HGNC:7673] | 6497 | -0.042 | -0.1515 | No |
| 63 | MMS19 | "MMS19 homolog, cytosolic iron-sulfur assembly component [Source:HGNC Symbol;Acc:HGNC:13824]" | 6733 | -0.058 | -0.1686 | No |
| 64 | NR0B1 | nuclear receptor subfamily 0 group B member 1 [Source:HGNC Symbol;Acc:HGNC:7960] | 6885 | -0.070 | -0.1788 | No |
| 65 | SRC | "SRC proto-oncogene, non-receptor tyrosine kinase [Source:HGNC Symbol;Acc:HGNC:11283]" | 6940 | -0.075 | -0.1814 | No |
| 66 | JMJD1C | jumonji domain containing 1C [Source:HGNC Symbol;Acc:HGNC:12313] | 6989 | -0.079 | -0.1833 | No |
| 67 | NCOA6 | nuclear receptor coactivator 6 [Source:HGNC Symbol;Acc:HGNC:15936] | 7171 | -0.093 | -0.1954 | No |
| 68 | MED4 | mediator complex subunit 4 [Source:HGNC Symbol;Acc:HGNC:17903] | 7236 | -0.098 | -0.1982 | No |
| 69 | CALR | calreticulin [Source:HGNC Symbol;Acc:HGNC:1455] | 7291 | -0.102 | -0.2001 | No |
| 70 | RXRA | retinoid X receptor alpha [Source:HGNC Symbol;Acc:HGNC:10477] | 7381 | -0.109 | -0.2046 | No |
| 71 | DCAF13 | DDB1 and CUL4 associated factor 13 [Source:HGNC Symbol;Acc:HGNC:24535] | 7459 | -0.115 | -0.2081 | No |
| 72 | LEF1 | lymphoid enhancer binding factor 1 [Source:HGNC Symbol;Acc:HGNC:6551] | 7467 | -0.116 | -0.2060 | No |
| 73 | MYOD1 | myogenic differentiation 1 [Source:HGNC Symbol;Acc:HGNC:7611] | 7839 | -0.143 | -0.2318 | No |
| 74 | KDM3A | lysine demethylase 3A [Source:HGNC Symbol;Acc:HGNC:20815] | 8264 | -0.176 | -0.2610 | No |
| 75 | TRIP12 | thyroid hormone receptor interactor 12 [Source:HGNC Symbol;Acc:HGNC:12306] | 8290 | -0.178 | -0.2590 | No |
| 76 | NR1H4 | nuclear receptor subfamily 1 group H member 4 [Source:HGNC Symbol;Acc:HGNC:7967] | 8481 | -0.192 | -0.2695 | No |
| 77 | RNF6 | ring finger protein 6 [Source:HGNC Symbol;Acc:HGNC:10069] | 8493 | -0.193 | -0.2660 | No |
| 78 | NRIP1 | nuclear receptor interacting protein 1 [Source:HGNC Symbol;Acc:HGNC:8001] | 8524 | -0.195 | -0.2639 | No |
| 79 | CRY2 | cryptochrome circadian regulator 2 [Source:HGNC Symbol;Acc:HGNC:2385] | 8536 | -0.196 | -0.2603 | No |
| 80 | KDM1A | lysine demethylase 1A [Source:HGNC Symbol;Acc:HGNC:29079] | 8608 | -0.202 | -0.2613 | No |
| 81 | ASXL1 | ASXL transcriptional regulator 1 [Source:HGNC Symbol;Acc:HGNC:18318] | 8610 | -0.202 | -0.2568 | No |
| 82 | RARG | retinoic acid receptor gamma [Source:HGNC Symbol;Acc:HGNC:9866] | 8700 | -0.209 | -0.2591 | No |
| 83 | DCAF1 | DDB1 and CUL4 associated factor 1 [Source:HGNC Symbol;Acc:HGNC:30911] | 8765 | -0.213 | -0.2593 | No |
| 84 | TAF11 | TATA-box binding protein associated factor 11 [Source:HGNC Symbol;Acc:HGNC:11544] | 8860 | -0.222 | -0.2616 | No |
| 85 | DAXX | death domain associated protein [Source:HGNC Symbol;Acc:HGNC:2681] | 8926 | -0.226 | -0.2616 | No |
| 86 | SIRT1 | sirtuin 1 [Source:HGNC Symbol;Acc:HGNC:14929] | 8947 | -0.227 | -0.2580 | No |
| 87 | FKBP4 | FKBP prolyl isomerase 4 [Source:HGNC Symbol;Acc:HGNC:3720] | 9205 | -0.248 | -0.2725 | No |
| 88 | LCOR | ligand dependent nuclear receptor corepressor [Source:HGNC Symbol;Acc:HGNC:29503] | 9227 | -0.250 | -0.2685 | No |
| 89 | NCOA1 | nuclear receptor coactivator 1 [Source:HGNC Symbol;Acc:HGNC:7668] | 9270 | -0.253 | -0.2661 | No |
| 90 | CRX | cone-rod homeobox [Source:HGNC Symbol;Acc:HGNC:2383] | 9397 | -0.263 | -0.2700 | No |
| 91 | PAGR1 | PAXIP1 associated glutamate rich protein 1 [Source:HGNC Symbol;Acc:HGNC:28707] | 9401 | -0.264 | -0.2642 | No |
| 92 | GTF2H1 | general transcription factor IIH subunit 1 [Source:HGNC Symbol;Acc:HGNC:4655] | 9455 | -0.269 | -0.2623 | No |
| 93 | TACC2 | transforming acidic coiled-coil containing protein 2 [Source:HGNC Symbol;Acc:HGNC:11523] | 9581 | -0.281 | -0.2657 | No |
| 94 | TACC1 | transforming acidic coiled-coil containing protein 1 [Source:HGNC Symbol;Acc:HGNC:11522] | 9690 | -0.290 | -0.2676 | No |
| 95 | FOXH1 | forkhead box H1 [Source:HGNC Symbol;Acc:HGNC:3814] | 9884 | -0.310 | -0.2757 | No |
| 96 | TRIM24 | tripartite motif containing 24 [Source:HGNC Symbol;Acc:HGNC:11812] | 9927 | -0.314 | -0.2719 | No |
| 97 | HMGA1 | high mobility group AT-hook 1 [Source:HGNC Symbol;Acc:HGNC:5010] | 9966 | -0.318 | -0.2677 | No |
| 98 | SMARCA4 | "SWI/SNF related, matrix associated, actin dependent regulator of chromatin, subfamily a, member 4 [Source:HGNC Symbol;Acc:HGNC:11100]" | 10009 | -0.322 | -0.2637 | No |
| 99 | KDM4C | lysine demethylase 4C [Source:HGNC Symbol;Acc:HGNC:17071] | 10040 | -0.325 | -0.2587 | No |
| 100 | ACTN4 | actinin alpha 4 [Source:HGNC Symbol;Acc:HGNC:166] | 10472 | -0.370 | -0.2841 | No |
| 101 | PPARGC1A | PPARG coactivator 1 alpha [Source:HGNC Symbol;Acc:HGNC:9237] | 10504 | -0.374 | -0.2781 | No |
| 102 | GRIP1 | glutamate receptor interacting protein 1 [Source:HGNC Symbol;Acc:HGNC:18708] | 10949 | -0.424 | -0.3032 | No |
| 103 | MED1 | mediator complex subunit 1 [Source:HGNC Symbol;Acc:HGNC:9234] | 11146 | -0.450 | -0.3084 | Yes |
| 104 | CRY1 | cryptochrome circadian regulator 1 [Source:HGNC Symbol;Acc:HGNC:2384] | 11247 | -0.463 | -0.3058 | Yes |
| 105 | TMF1 | TATA element modulatory factor 1 [Source:HGNC Symbol;Acc:HGNC:11870] | 11328 | -0.475 | -0.3013 | Yes |
| 106 | NKX3-1 | NK3 homeobox 1 [Source:HGNC Symbol;Acc:HGNC:7838] | 11348 | -0.478 | -0.2920 | Yes |
| 107 | BRD8 | bromodomain containing 8 [Source:HGNC Symbol;Acc:HGNC:19874] | 11557 | -0.508 | -0.2968 | Yes |
| 108 | ZBTB7A | zinc finger and BTB domain containing 7A [Source:HGNC Symbol;Acc:HGNC:18078] | 11600 | -0.516 | -0.2884 | Yes |
| 109 | NR4A1 | nuclear receptor subfamily 4 group A member 1 [Source:HGNC Symbol;Acc:HGNC:7980] | 11709 | -0.535 | -0.2848 | Yes |
| 110 | EP300 | E1A binding protein p300 [Source:HGNC Symbol;Acc:HGNC:3373] | 11741 | -0.540 | -0.2750 | Yes |
| 111 | LATS1 | large tumor suppressor kinase 1 [Source:HGNC Symbol;Acc:HGNC:6514] | 11760 | -0.542 | -0.2641 | Yes |
| 112 | HIF1A | hypoxia inducible factor 1 subunit alpha [Source:HGNC Symbol;Acc:HGNC:4910] | 11833 | -0.556 | -0.2572 | Yes |
| 113 | DDX54 | DEAD-box helicase 54 [Source:HGNC Symbol;Acc:HGNC:20084] | 11969 | -0.587 | -0.2545 | Yes |
| 114 | NR4A2 | nuclear receptor subfamily 4 group A member 2 [Source:HGNC Symbol;Acc:HGNC:7981] | 12368 | -0.698 | -0.2699 | Yes |
| 115 | THRAP3 | thyroid hormone receptor associated protein 3 [Source:HGNC Symbol;Acc:HGNC:22964] | 12426 | -0.717 | -0.2581 | Yes |
| 116 | STRN | striatin [Source:HGNC Symbol;Acc:HGNC:11424] | 12429 | -0.717 | -0.2421 | Yes |
| 117 | NCOA3 | nuclear receptor coactivator 3 [Source:HGNC Symbol;Acc:HGNC:7670] | 12455 | -0.727 | -0.2276 | Yes |
| 118 | MED25 | mediator complex subunit 25 [Source:HGNC Symbol;Acc:HGNC:28845] | 12480 | -0.741 | -0.2127 | Yes |
| 119 | MED13 | mediator complex subunit 13 [Source:HGNC Symbol;Acc:HGNC:22474] | 12504 | -0.749 | -0.1976 | Yes |
| 120 | NR4A3 | nuclear receptor subfamily 4 group A member 3 [Source:HGNC Symbol;Acc:HGNC:7982] | 12543 | -0.769 | -0.1832 | Yes |
| 121 | KDM5D | lysine demethylase 5D [Source:HGNC Symbol;Acc:HGNC:11115] | 12595 | -0.793 | -0.1692 | Yes |
| 122 | ARID5A | AT-rich interaction domain 5A [Source:HGNC Symbol;Acc:HGNC:17361] | 12705 | -0.867 | -0.1582 | Yes |
| 123 | CEBPB | CCAAT enhancer binding protein beta [Source:HGNC Symbol;Acc:HGNC:1834] | 12804 | -0.973 | -0.1438 | Yes |
| 124 | DDX5 | DEAD-box helicase 5 [Source:HGNC Symbol;Acc:HGNC:2746] | 12834 | -1.038 | -0.1226 | Yes |
| 125 | TOB2 | "transducer of ERBB2, 2 [Source:HGNC Symbol;Acc:HGNC:11980]" | 12838 | -1.041 | -0.0993 | Yes |
| 126 | JUND | "JunD proto-oncogene, AP-1 transcription factor subunit [Source:HGNC Symbol;Acc:HGNC:6206]" | 12865 | -1.116 | -0.0761 | Yes |
| 127 | CTNNB1 | catenin beta 1 [Source:HGNC Symbol;Acc:HGNC:2514] | 12866 | -1.119 | -0.0508 | Yes |
| 128 | BAZ2A | bromodomain adjacent to zinc finger domain 2A [Source:HGNC Symbol;Acc:HGNC:962] | 12879 | -1.166 | -0.0254 | Yes |
| 129 | TAF1 | TATA-box binding protein associated factor 1 [Source:HGNC Symbol;Acc:HGNC:11535] | 12881 | -1.192 | 0.0015 | Yes |
Table: GSEA details [plain text format]

  

Fig 2: GOMF\_NUCLEAR\_RECEPTOR\_BINDING      
 Blue-Pink O' Gram in the Space of the Analyzed GeneSet

  

Fig 3: GOMF\_NUCLEAR\_RECEPTOR\_BINDING: Random ES distribution      
 Gene set null distribution of ES for **GOMF\_NUCLEAR\_RECEPTOR\_BINDING**
